# Hybrid ^18^F-Fluoroethyltyrosine PET and MRI with Perfusion to Distinguish Disease Progression from Treatment-Related Change in Malignant Brain Tumors: The Quest to Beat the Toughest Cases

**DOI:** 10.2967/jnumed.122.265149

**Published:** 2023-07

**Authors:** Nathaniel J. Smith, Tristan K. Deaton, Wendy Territo, Brian Graner, Andrew Gauger, Scott E. Snyder, Michael L. Schulte, Mark A. Green, Gary D. Hutchins, Michael C. Veronesi

**Affiliations:** 1 School of Medicine, Indiana University, Indianapolis, Indiana;; 2 Weldon School of Biomedical Engineering, Purdue University, West Lafayette, Indiana; and; 3 Indiana University–Purdue University, Indianapolis, Indiana

**Keywords:** malignant brain tumors, WHO CNS grade 3 or 4 glioma, glioblastoma, brain metastasis, amino acid PET, ^18^F-fluoroethyltyrosine (FET) PET

## Abstract

Conventional MRI has important limitations when assessing for progression of disease (POD) versus treatment-related changes (TRC) in patients with malignant brain tumors. We describe the observed impact and pitfalls of implementing ^18^F-fluoroethyltyrosine (^18^F-FET) perfusion PET/MRI into routine clinical practice. **Methods:** Through expanded-access investigational new drug use of ^18^F-FET, hybrid ^18^F-FET perfusion PET/MRI was performed during clinical management of 80 patients with World Health Organization central nervous system grade 3 or 4 gliomas or brain metastases of 6 tissue origins for which the prior brain MRI results were ambiguous. The diagnostic performance with ^18^F-FET PET/MRI was dually evaluated within routine clinical service and for retrospective parametric evaluation. Various ^18^F-FET perfusion PET/MRI parameters were assessed, and patients were monitored for at least 6 mo to confirm the diagnosis using pathology, imaging, and clinical progress. **Results:** Hybrid ^18^F-FET perfusion PET/MRI had high overall accuracy (86%), sensitivity (86%), and specificity (87%) for difficult diagnostic cases for which conventional MRI accuracy was poor (66%). ^18^F-FET tumor-to-brain ratio static metrics were highly reliable for distinguishing POD from TRC (area under the curve, 0.90). Dynamic tumor-to-brain intercept was more accurate (85%) than SUV slope (73%) or time to peak (73%). Concordant PET/MRI findings were 89% accurate. When PET and MRI conflicted, ^18^F-FET PET was correct in 12 of 15 cases (80%), whereas MRI was correct in 3 of 15 cases (20%). Clinical management changed after 88% (36/41) of POD diagnoses, whereas management was maintained after 87% (34/39) of TRC diagnoses. **Conclusion:** Hybrid ^18^F-FET PET/MRI positively impacted the routine clinical care of challenging malignant brain tumor cases at a U.S. institution. The results add to a growing body of literature that ^18^F-FET PET complements MRI, even rescuing MRI when it fails.

## Visual Abstract

**Figure:**
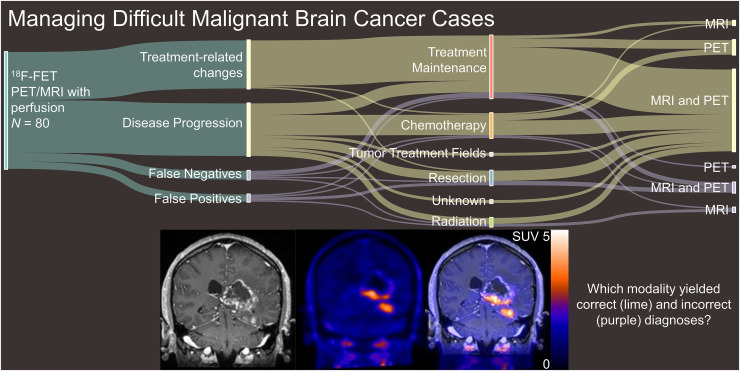

Malignant gliomas of World Health Organization central nervous system grade 3 or 4 (adult-type diffuse glioma, ATDG) and brain metastases (BM) cause significant morbidity and mortality annually (1). Although management has improved, these malignancies remain difficult to treat. Even with specialized care, patients with glioblastoma have a mean survival of 15–20 mo with standard-of-care therapy, and only about 5% survive past 5 y (2). BMs are 10 times more common than primary malignant brain tumors, portend a poor prognosis, and continue rising in incidence (3*,*^4^).

Conventional MRI is the standard clinical imaging modality for managing brain tumors; however, it remains suboptimal for response assessment and treatment monitoring when distinguishing the progression of disease (POD) from treatment-related changes (TRCs) (5). MRI signal abnormalities lack biologic specificity, as T2-derived abnormalities reflect tissue water content, and contrast enhancement identifies regions of high blood–brain barrier permeability.

In MRI, perfusion-weighted imaging (PWI) indirectly measures malignancy by detecting neovascularity. Dynamic susceptibility contrast PWI captures signal loss within susceptibility-weighted sequences as paramagnetic gadolinium moves through tumor tissue (6). Dynamic contrast-enhanced PWI evaluates T1 relaxivity as gadolinium contrast medium passes through tissue (7). Although PWI techniques partially overcome conventional MRI limitations, reported clinical thresholds vary widely because of differences in acquisition protocols, scanner hardware, and overlapping tumor and normal-tissue parametric distributions (8*–*11).

To help overcome MRI’s limitations, international working groups recommend amino acid PET imaging for complementary assessment of malignant brain tumors given superior tumor-to-background contrast (12*–*14). ^18^F-fluoroethyltyrosine (^18^F-FET) is the most commonly used amino acid radiotracer, providing a high diagnostic value for differentiating POD from TRC (15*–*18). PWI combined with ^18^F-FET PET demonstrates increased sensitivity and specificity for delineating POD from TRC in malignant brain tumors, with hybrid ^18^F-FET PET/MRI further increasing the accuracy (5*,*^19^*,*^20^).

We report the clinical application of hybrid ^18^F-FET PET/MRI to patients with malignant brain tumors at a U.S. institution to discern POD from TRC. This report provides additional evidence supporting the complementary nature of ^18^F-FET PET and MRI, reinforcing the European Association of Neurooncology/Response Assessment in Neurooncology (EANO/RANO) working group recommendations.

## MATERIALS AND METHODS

**FIGURE 1.**
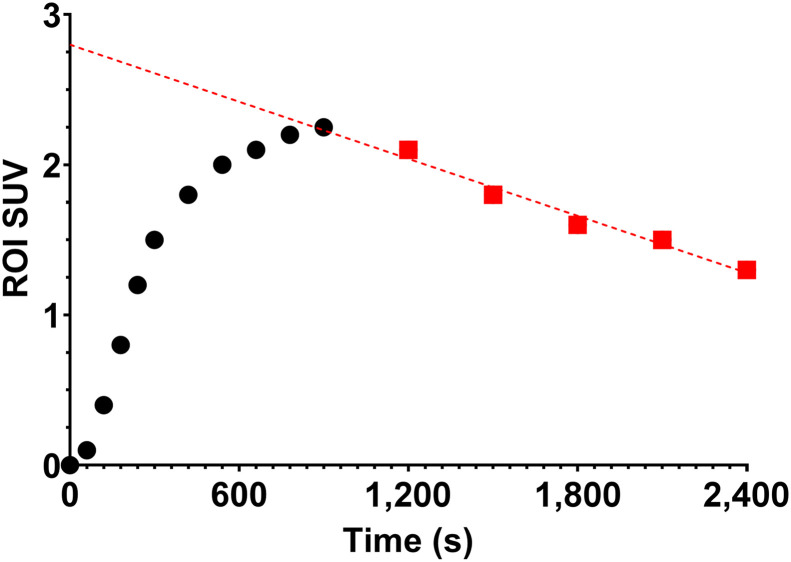
Dynamic slope and intercept estimation method. Regions of interest (ROIs) were located by neuroradiologist, and SUV_mean_ was extracted for every frame in dynamic sequence. Linear regression to scan’s final 20–40 min determined slope and intercept terms. Slope is reported in units of SUV/h. For TBR analysis, ROIs were normalized by contralateral reference tissue ROI for every frame in dynamic sequence with same linear regression procedure. TTP was estimated from SUV dynamic sequence global maximum.

^18^F-FET PET/MRI (3T Biograph mMR; Siemens) was performed for 80 adult patients with known ATDG (*n* = 42) or BM (*n* = 38). Advanced imaging discerned whether abnormalities in standard-of-care imaging corresponded to POD or TRC, with a 6-mo clinical follow-up (33 BM and 36 ATDG) or pathologic reference (5 BM and 6 ATDG) diagnosis. The institutional review board approved this study, and all subjects provided written informed consent for imaging with ^18^F-FET, which was prepared and clinically administered under expanded-access investigational-new-drug application 150883. Patients underwent standard cranial MRI, including 3-dimensional T1-weighted sequences before and after contrast medium, T2-weighted sequences, 3-dimensional fluid-attenuated inversion recovery sequences, diffusion-weighted imaging, apparent diffusion coefficient imaging, and susceptibility-weighted imaging. Patients received a half-and-half gadobutrol (Gadavist [Bayer]; 0.1 mL/kg of body weight) injection before dynamic susceptibility contrast MRI (repetition time, 1,600 ms; echo time, 30.0 ms; 90° flip angle; 1.7 × 1.7 × 4.0 mm voxel size; 2,020-ms temporal resolution) and dynamic contrast-enhanced TWIST (time-resolved angiography with interleaved stochastic trajectories; Siemens) MRI (repetition time, 3.91 ms; echo time, 1.54 ms; dynamic temporal resolution, 2.70 s; 10° flip angle; 1.1 × 1.1 × 5.0 mm voxel size). ^18^F-FET PET data were acquired in list mode from 0 to 40 min, concurrent with MRI acquisitions, allowing reconstruction as both single-frame late static images and a dynamic sequence for assessing regional radiopharmaceutical kinetics. ^18^F-FET (503–810 MBq) was administered as a bolus followed by a saline flush. Relatively high ^18^F-FET doses were used to enhance small-lesion (<10 mm) detection, with estimated critical organ doses remaining commensurate with standard clinical nuclear medicine procedures. Image analysis and interpretation followed the Society of Nuclear Medicine and European Association of Nuclear Medicine procedural recommendations for ^18^F-FET PET/MRI of brain tumors (14). ^18^F-FET analysis parameters included static (mean, maximum) and dynamic (slope, intercept, time-to-peak [TTP]) assessment for SUV and tumor-to-brain ratio (TBR) metrics (Fig. 1). PWI parameters included relative cerebral blood volume (rCBV), capillary permeability volume transfer constant (K_trans_), and extravascular extracellular volume fraction. The Supplemental Methods contain more complete image acquisition, processing, and analysis details (supplemental materials are available at http://jnm.snmjournals.org) (7*,*^14^*,*^21^*–*32).

## RESULTS

### Patients

Eighty patients (47 men, 33 women) aged 17–77 y underwent ^18^F-FET PET/MRI. Most patients were at least 50 y old (75%) and were Caucasian (91%) (Table 1). Standard-of-care treatments included MRI-localized gross (36/80) and subtotal (16/80) resection; stereotactic radiosurgery (56/80); γ-knife (13/80) and whole-brain radiation (9/80); and temozolomide (32/80), bevacizumab (10/80), monoclonal antibody (28/80), DNA alkylation (15/80), and small-molecule kinase inhibitor (5/80) chemotherapies. Equivocal MRI examinations occurred a median of 188 d (range, 52–1,252 d) after the initiation of radiotherapy, and patients received a median 4 adjuvant doses (range, 0–12) of temozolomide. ^18^F-FET PET/MRI was performed a median of 10 mo (range, 2–95 mo) after radiation treatment, 9 mo (range, 1–76 mo) after surgery, and 38 d (range, 10–394 d) after the latest MRI exam. The supplemental materials contains additional patient information.

**TABLE 1.** Demographics for the 80 Study Patients

Demographic	*n*
Sex	
Male	47 (59%)
Female	33 (41%)
Age (y)	
<40	6 (7.5%)
40–49	14 (18%)
50–59	29 (36%)
60–69	25 (31%)
≥70	6 (7.5%)
White/Caucasian*	73 (91%)
Black/African American	3 (4%)
Hispanic/Latinx	2 (3%)
Asian	2 (3%)

* Denotes overrepresentation relative to overall US incidence.

Forty-two ATDG patients (30 glioblastoma, 2 grade 4 diffuse astrocytoma, and 10 grade 3 diffuse astrocytoma) underwent ^18^F-FET PET/MRI a median of 47 d after brain MRI with equivocal findings. Isocitrate dehydrogenase was wild type in 79% (33/42) patients, mutated in 19% (8/42) patients, and unknown in 1 patient. (6)-methylguanine-DNA methyltransferase was unmethylated in 43% (18/42) of patients, low-level methylated in 5% (2/42) of patients, methylated in 24% (10/42) of patients, hypermethylated in 14% (6/42) of patients, and unknown in 6 patients. Thirty-eight patients underwent ^18^F-FET PET/MRI to assess BM treatment response. Disease origins included lung in 47% (18/38), colon or rectum in 5% (2/38), kidney in 13% (5/38), melanoma in 11% (4/38), breast in 18% (7/38), and thyroid in 3% (1/38) and were unspecified in 3% (1/38).

### Observed Performance of ^18^F-FET PET/MRI

**FIGURE 2.**
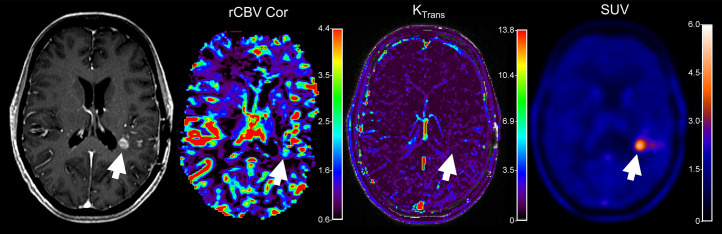
Patient with suspected recurrence of left parietal glioma (arrows) of World Health Organization central nervous system grade 4. Patient was initially evaluated with ^18^F-FET PET/MRI for increasing contrast-enhanced T1-weighted MRI findings posterior to left resection cavity. Dynamic contrast-enhanced K_trans_ parameter met threshold for progression (0.38), but maximum rCBV value remained borderline (3.0). ^18^F-FET PET TBR_max_ and TBR_mean_ exceeded threshold at 2.75 and 2.3, respectively, supporting diagnosis of disease progression. Bevacizumab treatment was initiated, and enhancement and perfusion pattern improved on follow-up MRI, remaining stable for 2.5 y. Serial ^18^F-FET PET/MRI 2 y later remained stable.

Table 2 summarizes the institutional diagnostic performance of ^18^F-FET PET/MRI. The accuracy, sensitivity, and specificity were similar across all disease origins (∼85%). Figure 2 demonstrates an example case with hybrid ^18^F-FET PET/PWI assisting to provide a diagnosis, and Supplemental Figures 1–4 provide examples of true-positive, true-negative, false-positive, and false-negative institutional diagnoses. Only 11 cases (14%) were misidentified in this study, 5 as false-positives and 6 as false-negatives. False-negative diagnoses occurred nearly twice as frequently in ATDG patients (4/42, 9.5%) as in BM patients (2/38, 5.3%). Of the 11 misdiagnosed patients, 7 had a tumor volumes of less than 10 cm^3^. Most mischaracterized lesions had increased in size from the previous MRI exam (7/11); 3 were stable, and 1 decreased in size.

**TABLE 2.** Institutional Performance with Hybrid ^18^F-FET PET/MRI

Index	Overall	High-grade glioma	BM
Total patients	80	42	38
True-positive (true POD)*	36	28	8
True-negative (true TRC)*	33	8	25
False-positive*	5	2	3
False-negative*	6	4	2
Accuracy	86%	86%	87%
Sensitivity	86%	88%	80%
Specificity	87%	83%	88%
Positive predictive value	88%	93%	73%
Negative predictive value	85%	67%	93%
Positive likelihood ratio	6.5	4.4	7.5
Negative likelihood ratio	0.16	0.16	0.22
False-positive rate	13%	20%	11%
False-negative rate	14%	13%	20%

* Confirmed by 6 mo of follow-up.

Performance shown demonstrates overall clinical performance with integrating ^18^F-FET PET/MRI into care, using literature-based and institutional thresholds (rCBV > 3:1, K_trans_ > 0.26, TBR_max_ > 2.5) to assist in image-derived diagnosis.

**FIGURE 3.**
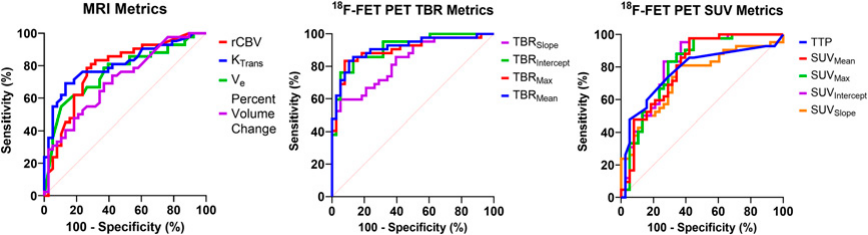
ROC curve analysis of perfusion-weighted MRI and ^18^F-FET PET/MRI. Sensitivity was calculated as ratio of detected cases to all POD cases, and specificity was calculated as ratio of detected cases to all TRC cases. MRI metrics include rCBV, K_trans_, and extravascular extracellular volume fraction (V_e_). PET metrics included TBR, SUV, and TTP concentration. Dashed red line represents nondiagnostic test performance. Associated ROC quantitative analyses are in Table 3.

Figure 3 and Supplemental Table 1 display the combined pathology receiver-operating-characteristic (ROC) performance for all perfusion MRI, TBR normalized ^18^F-FET PET/MRI uptake, and standardized ^18^F-FET PET/MRI uptake parameters. The supplemental materials contain replicate ROC analyses stratified by disease origin (BM or ATDG), optimized cutoff thresholds, performance characteristics, and statistical justification. Increasing tumor volume on conventional MRI was only 60% (33/55) predictive of POD, whereas stagnant or receding MRI tumor volume was 64% (16/25) predictive of TRC. Overall, ^18^F-FET PET/MRI metrics met or exceeded the diagnostic performance of perfusion MRI metrics. For combined ATDG and BM patients, maximum, mean, and intercept TBR metrics (TBR_max_, TBR_mean_, and TBR_intercept_, respectively) generated area-under-the-ROC-curve (AUC) performance at or above 0.90 (Table 3). Institutional accuracy values mirrored the performance of these 3 metrics within 2% across all disease origins (BM or ATDG). In contrast, no perfusion-weighted MRI metric demonstrated an accuracy above 76%.

**TABLE 3.** Retrospective ROC-Optimized Thresholds and Analysis Results in All Patients (38 TRC, 42 POD) (for Fig. 3)

Modality	Parameter	Cutoff (%)	SN (%)	SP (%)	ACC (%)	PPV (%)	NPV (%)	AUC	95% CI	Adjusted *P*
MRI	Volume change	>13.5	69	63	66	67	65	0.71	0.60–0.82	<0.01
Perfusion MRI	rCBV	>3.85	79	74	76	77	76	0.78	0.67–0.88	<0.0001
	K_trans_	>0.58	76	76	76	78	74	0.81	0.71–0.90	<0.0001
	V_e_	>0.98	62	82	71	79	66	0.76	0.65–0.87	<0.0001
^18^F-FET PET (TBR)	TBR_slope_	<−0.69	67	79	73	78	68	0.83	0.74–0.92	<0.0001
	TBR_intercept_	>2.39	83	87	85	88	83	0.91	0.85–0.98	<0.0001
	TBR_max_	>2.69	83	92	88	92	83	0.90	0.84–0.97	<0.0001
	TBR_mean_	>2.16	86	87	86	88	85	0.91	0.85–0.98	<0.0001
^18^F-FET PET (SUV)	SUV_slope_	<0.24	81	66	73	71	75	0.74	0.63–0.85	<0.001
	SUV_intercept_	>2.07	83	74	79	78	80	0.82	0.72–0.91	<0.0001
	SUV_max_	>2.42	83	71	76	76	77	0.81	0.71–0.91	<0.0001
	SUV_mean_	>2.01	81	66	74	72	76	0.80	0.70–0.90	<0.0001
	TTP	<1,800	74	71	73	74	71	0.78	0.67–0.88	<0.0001

SN = sensitivity; SP = specificity; ACC = accuracy; PPV = positive predictive value; NPV = negative predictive value; V_e_ = extravascular extracellular volume fraction.

Significance is adjusted for multiple comparisons using Benjamini–Hochberg method and tests for AUC > 0.5. All metrics tested as significant. Cutoffs were calculated by optimizing geometric mean of sensitivity and specificity for ROC curve.

**FIGURE 4.**
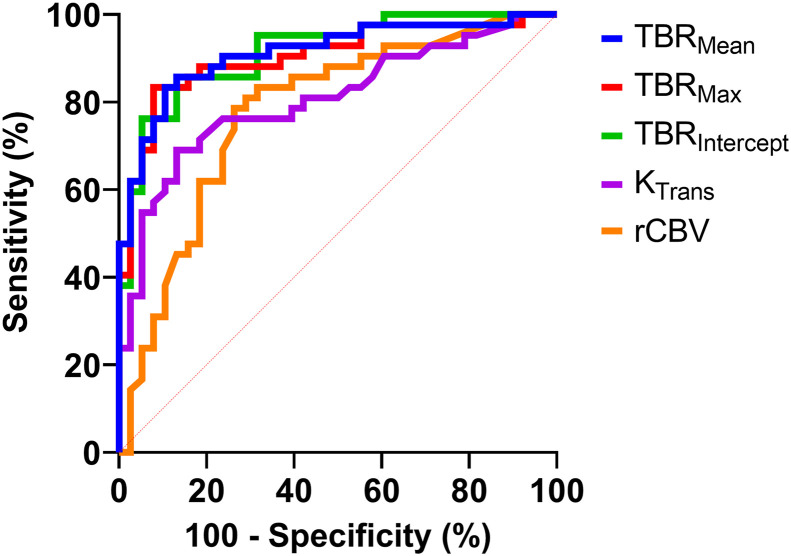
ROC curve performance comparison for select perfusion-weighted MRI and ^18^F-FET PET parameters. Sensitivity was calculated as ratio of detected cases to all POD cases, and specificity was calculated as ratio of detected cases to all TRC cases. MRI metrics include rCBV and K_trans_. PET metrics included TBR_intercept_, TBR_max_, and TBR_mean_. Dashed red line represents nondiagnostic test performance. Associated comparative tests are in Table 4.

When directly compared, TBR_max_, TBR_mean_, and TBR_intercept_ performed significantly better than K_trans_ and rCBV (Fig. 4; Table 4). The optimal perfusion-weighted MRI POD threshold (rCBV > 3.85) would have led to 10 false-positive and 9 false-negative mischaracterizations for this cohort without ^18^F-FET PET. In these instances, 7 false-positives and 5 false-negatives would have been corrected by combination with the TBR_max_ threshold of more than 2.69 or the TBR_mean_ threshold of more than 2.16. Alternatively, if a TBR_max_ of more than 2.69 was the sole diagnostic metric, 7 false-negative and 3 false-positive cases would have occurred, and only 3 of the false-negative diagnoses would have been corrected by the rCBV threshold of more than 3.85.

**TABLE 4.** ROC Comparison of Select Perfusion-Weighted MRI and ^18^F-FET PET/MRI Metrics (for Fig. 4)

Metrics compared	Metric 1 AUC	Metric 2 AUC	Adjusted *P*
TBR_intercept_ vs. rCBV	0.9148	0.7785	0.024*
TBR_mean_ vs. rCBV	0.9123	0.7785	0.015*
TBR_max_ vs. rCBV	0.9041	0.7785	0.013*
TBR_intercept_ vs. K_trans_	0.9148	0.8058	0.025*
TBR_mean_ vs. K_trans_	0.9123	0.8058	0.026*
TBR_max_ vs. K_trans_	0.9041	0.8058	0.028*
K_trans_ vs. rCBV	0.8058	0.7785	0.310 (NS)

* *P* < 0.05.

NS = not significant.

*P* values are calculated using Hanley ROC comparison method and adjusted for multiple comparisons using Benjamini–Hochberg method. Significance tests are for overperformance of metric 1 relative to metric 2. Evaluated over entire 80-patient sample, listed TBR metrics demonstrate superior performance in AUC compared with K_trans_ and rCBV. No significant difference in performance exists between rCBV (dynamic susceptibility contrast MRI) and K_trans_ (dynamic contrast-enhanced MRI).

When stratified by disease origin, select dynamic ^18^F-FET PET/MRI metrics generated strong retrospective performance. Slope TBR metrics (TBR_slope_) were 95% (36/38) accurate with an AUC of 0.97 for BM patients (Supplemental Fig. 5; Supplemental Table 2), and intercept SUV metrics (SUV_intercept_) were 95% (40/42) accurate with an AUC of 0.96 for ATDG patients (Supplemental Fig. 6; Supplemental Table 3). Of the 5 BM patients who were incorrectly diagnosed, 4 had TBR_slope_ values that were consistent with their correct diagnosis. Perfusion-weighted MRI parameters yielded up to 90% (38/42) accuracy for ATDG patients, and 5 of 6 of the misdiagnosed ATDG patients had K_trans_ values and extravascular extracellular volume fractions consistent with their correct diagnosis. Further details on all patient disease characteristics, treatments, and imaging parameters can be found in Supplemental Tables 4–11. Supplemental Table 12 includes a small subset analysis of interrater reliability for PWI parameters, indicating strong correspondence between readers for K_trans_ and rCBV.

## DISCUSSION

This study demonstrated the utility of hybrid ^18^F-FET PET/MRI with PWI in routine clinical medicine, applied across various primary and secondary disease origins, treatment regimens, and diagnostic timelines. Despite the patient heterogeneity, reported PWI and ^18^F-FET PET performance were consistent with the literature. For ATDG patients, our rCBV (DSC MRI) sensitivity of 84%, specificity of 80%, and AUC of 0.88 were within the confidence bounds of a recent metaanalysis released by Fu et al. (33). Additionally, the K_trans_ (dynamic contrast-enhanced MRI) specificity of 80% and AUC of 0.88 for ATDG patients were within the confidence bounds of a metaanalysis released by Taylor et al., whereas our reported sensitivity was slightly higher, at 94% (34). Evidence for PWI is limited in BM patients, but Cicone et al. reported an rCBV accuracy of 76% and AUC within 0.65–0.96, which complement our 76% accuracy and AUC of 0.74 (35).

The complementary nature of perfusion-weighted MRI and amino acid PET uptake imaging improved clinician confidence compared with MRI alone. Importantly, when dynamic susceptibility contrast or dynamic contrast-enhanced MRI is suboptimal or nondiagnostic, ^18^F-FET PET still supports high clinician diagnostic confidence because it resists PWI failure (36). Because interreader qualitative assessment varies with PWI alone (37), complementary imaging enables a more robust diagnostic outcome. Previous studies have advocated for sequential PWI and ^18^F-FET PET in glioma evaluation due to a 100% reported positive predictive value for rCBV, but our reported 93% positive predictive value for rCBV did not exceed that of TBR_mean_ or TBR_max_ (96% and 97%, respectively) (38). ^18^F-FET PET parameters also provided higher accuracy (TBR_mean_, 86%; TBR_max_, 88%) than rCBV (83%), supporting the use of simultaneous hybrid imaging, when available.

In this study, diagnostic accuracy was 89% (58/65) when PWI and ^18^F-FET PET findings were concordant. When discordant, ^18^F-FET PET indicated the correct diagnosis in 80% (12/15) of patients. However, 45% (5/11) of the false diagnoses occurred when hybrid imaging was discordant. In the 20% (3/15) of discordant imaging findings for which PWI indicated the correct diagnosis, ^18^F-FET PET failed to detect POD. Overall, the institutional diagnostic accuracy mirrors that of ^18^F-FET PET alone. Hybrid imaging exceeded the performance of perfusion MRI alone, but the impact of other useful MRI techniques, such as diffusion-weighted imaging, MR spectroscopy, and kurtosis imaging, was not evaluated in this study (19*,*^39^*–*41).

Our institution’s ^18^F-FET PET criteria included a TBR_max_ of more than 2.5 and a TBR_mean_ of more than 2.0, guided by publications cited in the EANO/RANO update (14). Calculated glioma thresholds included a TBR_max_ of more than 2.42 and a TBR_mean_ of more than 2.16, in support of the criteria. Individually, these static ^18^F-FET PET TBR parameters achieved 86%–88% diagnostic accuracy, 84%–88% sensitivity, and 90% specificity, consistent with other reports (17*,*^39^). For BM, our diagnostic accuracy with TBR_max_ approximates literature values, but with an elevated threshold of 2.79 relative to the 2.55 literature threshold (15*,*^16^). POD prevalence was only 26% (10/38) in our BM patients, which could generate a biased threshold estimate. Combined, our data support a TBR_max_ threshold of 2.69 (88% accuracy, 83% sensitivity, 92% specificity) across all disease origins. Our threshold exceeds the suggested EANO/RANO criteria of 2.5 (84% accuracy, 86% sensitivity, 82% specificity), reflecting a blend of the BM and ATDG stratified criteria. Compared with international recommendations, our threshold improves the overall accuracy for our patients, but at the cost of slightly reduced sensitivity and an increased false-negative rate.

Guidelines for ATDG and BM evaluation recommend the use of static and dynamic ^18^F-FET PET analysis (13*,*^14^), promoting slope SUV metrics (SUV_slope_) and TTP as the most accurate dynamic metrics. Although SUV_slope_ and TTP demonstrated strong performance for BM and ATDG, with our results matching literature accuracy (39), our data suggest the need for an origin-specific diagnostic approach. For ATDG, SUV_intercept_ enabled a 95% diagnostic accuracy, with an AUC greater than the TTP and SUV_slope_ (Supplemental Table 3). Because the SUV_intercept_ varies with the TTP, SUV_slope_, and the absolute SUV scaling of the time–activity curve, we hypothesize that performance characteristics can be attributed to this multifactorial diagnostic combination. For BM, TBR_slope_ enabled a 95% diagnostic accuracy (Supplemental Table 2), outperforming SUV_slope_. Because TBR metrics normalize the SUV signal to a contralateral reference region, we posit that TBR_slope_ achieves high accuracy by reducing the impact of regional uptake differences and patient weight variability.

^18^F-FET prompted false-negative conclusions more frequently when the lesion was less than 10 cm^3^ in volume (Supplemental Fig. 4), consistent with other reports indicating the importance of suprathreshold tumor volumes (42). Partial-volume artifacts can dilute the ^18^F-FET signal from small lesions, obscuring them with adjacent brain signals. To address this, serial ^18^F-FET PET/MRI may enhance subclinical lesion interpretation when compared with single-session ^18^F-FET PET/MRI and periodic follow-up PWI.

This study demonstrates the value of clinical ^18^F-FET PET/MRI; however, there are still limitations to its clinical use. Clinical scans are inconsistent in their posttreatment timing and are subject to treatment heterogeneity. This study is also susceptible to referral bias, omitting cases in which a prior MRI exam sufficiently established a definitive diagnosis, as evidenced by the low diagnostic performance of contrast-enhanced MRI lesion volume change relative to other investigated parameters (Table 3). However, this implicit focus on challenging case referrals is consistent with other reports in the literature and with the common clinical indications for ^18^F-FET PET (43). Additionally, this study was limited in sample size for stratification by disease type. When assessing ATDG alone, there was an asymmetrically higher number of cases of POD (76%, 32/42) than of METs (24%, 10/42). Conversely, there were fewer METs with POD (26%, 10/38) than with TRC (74%, 28/38). Although most analyses were considered in aggregate, the supplemental materials stratified by disease type did not have balanced outcomes and should be interpreted cautiously.

## CONCLUSION

This study demonstrates the overall benefit of implementing hybrid ^18^F-FET PET/MRI for patients with malignant brain tumors when conventional MRI and PWI were equivocal for discerning disease progression from nonmalignant treatment changes. Sample size limitations and the subjective nature of regional analysis may limit the generalizability of the study findings; however, this study provides a benchmark for prospective analyses and establishes a framework through which ^18^F-FET PET/MRI may be clinically introduced.

## DISCLOSURE

No potential conflict of interest relevant to this article was reported.

## Notes

KEY POINTS **QUESTION:** What is the clinical impact of perfusion ^18^F-FET PET/MRI on assessing posttreatment brain malignancies? **PERTINENT FINDINGS:** TBR metrics can distinguish posttreatment disease progression from treatment-related change with high accuracy. **IMPLICATIONS FOR PATIENT CARE:** ^18^F-FET PET/MRI improves clinician diagnostic confidence and informs timely, appropriate treatment modifications.
